# Near-Infrared Hyperspectral Imaging Pipelines for Pasture Seed Quality Evaluation: An Overview

**DOI:** 10.3390/s22051981

**Published:** 2022-03-03

**Authors:** Priyanka Reddy, Kathryn M. Guthridge, Joe Panozzo, Emma J. Ludlow, German C. Spangenberg, Simone J. Rochfort

**Affiliations:** 1 Agriculture Victoria, AgriBio, Centre for AgriBioscience, Bundoora, VIC 3083, Australia; priyanka.reddy@agriculture.vic.gov.au (P.R.); kathryn.guthridge@agriculture.vic.gov.au (K.M.G.); emma.ludlow@agriculture.vic.gov.au (E.J.L.); german.spangenberg@agriculture.vic.gov.au (G.C.S.); 2 Agriculture Victoria Research, 110 Natimuk Road, Horsham, VIC 3400, Australia; joe.panozzo@agriculture.vic.gov.au; 3 Centre for Agriculture Innovation, University of Melbourne, Parkville, VIC 3010, Australia; 4 School of Applied Systems Biology, La Trobe University, Bundoora, VIC 3083, Australia

**Keywords:** multispectral imaging, NIR-HSI, quality evaluation, chemometrics, seed quality, perennial ryegrass, tall fescue, *Lolium* spp.

## Abstract

Near-infrared (800–2500 nm; NIR) spectroscopy coupled to hyperspectral imaging (NIR-HSI) has greatly enhanced its capability and thus widened its application and use across various industries. This non-destructive technique that is sensitive to both physical and chemical attributes of virtually any material can be used for both qualitative and quantitative analyses. This review describes the advancement of NIR to NIR-HSI in agricultural applications with a focus on seed quality features for agronomically important seeds. NIR-HSI seed phenotyping, describing sample sizes used for building high-accuracy calibration and prediction models for full or selected wavelengths of the NIR region, is explored. The molecular interpretation of absorbance bands in the NIR region is difficult; hence, this review offers important NIR absorbance band assignments that have been reported in literature. Opportunities for NIR-HSI seed phenotyping in forage grass seed are described and a step-by-step data-acquisition and analysis pipeline for the determination of seed quality in perennial ryegrass seeds is also presented.

## 1. Introduction

Near-infrared spectroscopy (NIRS) is a widely used technique for performing quality control in laboratories that are associated with pharmaceutical [1], petroleum [2] and agriculture. Now commonly used for various applications, NIR, which absorbs at wavelengths of 800–2500 nm (12,500–4000 cm^−1^) of the electromagnetic spectrum, was developed by Karl Norris, United States Department of Agriculture (USDA), in the 1960s for the purposes of quality assessment of agricultural products [3,4]. In 1962, Norris was already ahead of his time, using NIR data to build calibration models using advanced statistical methods [5,6,7]. The use of NIR has since been extended to applications such as quality assurance of agricultural products. For example, the dairy industry uses NIRS to perform measurements of lactose, protein and fat in milk [8,9,10]. There are also numerous reports of NIRS used in the grains industry, for example the discrimination among cultivars of wheat kernels [11], detection of fungal infestation [12] and prohibited additives [13,14]. Plant stress [15] and nutritive value [16] can also be measured using portable NIR spectrometers in the field for plant breeding programs. The high-throughput nature of these phenotyping imaging tools has allowed large-scale implementation in breeding by genomic selection [17] in agriculture. NIRS is also widely used in the meat industry for applications such as classification of poultry carcasses infected with disease in real time [18,19] and determination of sensory and texture characteristics of beef [20], as well as meat properties and chemical composition [20]. Applications in plant-based industries include fruit, grain and seed quality, particularly pathogen infestation and varietal purity, as well as chemical composition. The list of applications continues to grow rapidly [21,22,23,24,25,26,27,28,29,30,31,32,33,34,35,36,37].

The main advantage of NIRS is that the methodology is non-destructive and high throughput. NIRS platforms provide spectral information, usually an average of a few selected points or the mean spectrum of a larger area. However, this is not so useful when objects are non-uniform and the features of interest are restricted to a relatively small but unknown part of the object [38]. When NIRS is coupled to imaging techniques, such as multispectral and hyperspectral techniques, both spatial and spectral information can be obtained. NIRS-multispectral imaging captures images within a small number of spectral bands, usually a maximum of 10 [39]. NIR-hyperspectral imaging (NIR-HSI), an extension of NIRS-multispectral imaging, captures hundreds of contiguous wavelength bands in the NIR region for each pixel. Coupling NIRS with an imaging technique was first introduced in the late 1990s [40] and the technology has advanced such that it is now routinely applied in fields such as military surveillance [41], astronomy [42], environmental monitoring [43] and agriculture.

The non-destructive nature of NIRS imaging allows quality control in agriculturally important fruit (e.g., apples, peaches and apricots) to be obtained, [44,45,46,47,48] particularly for detecting external defects. Typically, fresh fruits are graded into categories based on parameters that affect quality, such as external defects, size, shape and colour, to comply with the Organisation for Economic Co-operation and Development (OECD) standards [49]. External defects are the most difficult to distinguish in automated systems; however, with the advent of NIRS imaging and, more recently, hyperspectral imaging, applications for detecting surface defects in fruit have been rapidly increasing. For example, NIRS and hyperspectral models for detecting surface defects originating from fungal infestation, insect damage on oranges [50], mangoes [51] and peaches [46], as well as damaged almond nut [52], have been reported. More recently, fruit ripening and maturity are also being assessed using NIRS imaging such as ripeness in grapes and firmness and maturity in lime and mango by determination of the total soluble solid and titratable acidity [53]. NIRS for sorting fruit based on sweetness is in use in more than 1000 packing houses in Japan using single-point measurements [54] and conceivably superseded with developments driven in NIR imaging as a visualization technique for sugar content and distribution [54,55,56,57].

It has become increasingly evident that hyperspectral imaging can be applied to the non-destructive qualitative and quantitative determination of the desired features of selected samples, without contact [58]. Thus, it is very much suited for routine diagnostics such as food quality assessments and safety analyses [59]. A further advantage is that the NIR-HSI spectrum is collected at each pixel in the image, providing both the distribution and chemical composition of individual components [58], whereas NIRS only acquires a single spectrum for the sample [40]. This makes NIR-HSI suitable for heterogenous samples or for exploratory analyses, where the composition of the sample is largely unknown [38]. Despite the time and financial investment required for method development and the large data sets obtained, once a method is developed, it is inexpensive and routine with the benefits of reduced labour, turnaround and cost, compared to traditional methods used for inspecting and testing agricultural food products [60,61].

Recently, there have been numerous studies reporting on use of NIR-HSI for grain and seed quality [62], varietal purity [63], pathogen detection, seed constituents [64] and viability [65]. While there are various reviews on seed applications, there are currently no detailed data-acquisition and data-analysis pipelines provided for hyperspectral imaging for pasture seeds. For example, perennial ryegrass seeds possess a complex morphology that is naturally colonized by endophytic fungus which forms a mutualistic association. This review explores similar applications of hyperspectral imaging in agriculture with a focus on seed quality applications. Here, we provide a detailed summarization of the challenges and requirements of acquiring *Lolium* species, such as perennial ryegrass seed hyperspectral data for determining key seed quality attributes.

## 2. Hyperspectral Imaging Instruments

The components, configuration and design of a hyperspectral system are essential in the acquiring of reliable and high-quality images and data. A typical hyperspectral imaging system consists of a light source, wavelength dispersion device and an area detector (camera).

HSI systems are generally either active or passive systems. Active HSI systems are equipped with an active light source, such as those described in Table 1. A passive HSI system relies on ambient radiation, e.g., sunlight [66]. The light source is an important aspect in the excitation of the target sample and the quality of the images strongly depends on a well-balanced light intensity. Light sources include halogen lamps, light emitting diodes (LED), lasers and tuneable light sources. Fluorescent lamps are not recommended as there are inconsistencies in energy levels for different wavelengths [67].

Broadband light sources, including the halogen lamp, LED and tuneable light, are used with a wavelength dispersion device, such as a prism, grating, or filter, a key component in hyperspectral instruments [68]. It is placed between the detector and sample and it is used to disperse broadband light into different wavelengths. A filter is generally used for multispectral imaging systems whereas a prism and grating are widely used in hyperspectral systems. The final component of the system is the camera which has the role of quantifying the intensity of the light generated by the light source by converting photons into electrons. Charge-couple devices (CCDs) and complementary metal-oxide-semiconductors (CMOSs) are the two major cameras [68,69]. The CCD image sensor is superior to the CMOS device and generates high-quality image data.

The typical hyperspectral components utilised in the development of seed quality applications, include a halogen lamp light source, imaging spectrographs that covers VNIR, SW-NIR and NIR spectral ranges and, mostly, CCD-type image sensors.

**Table 1:** Advantages and disadvantages of using various light sources in hyperspectral imaging systems.

Light Source	Application	Advantages	Disadvantages	Example References
Halogen lamps	VIS NIR SW-NIR Broadband white light	Delivers smooth and continuous spectrum in the spectral range High light intensity	Short lifetime High heat Unstable ^1^ (operating voltage fluctuations) Sensitive to vibration	[70]
LED	From UV to SW-NIR, while some LEDs emit light from LW-NIR to MIR Broadband white light Excitation mode (fluorescence)	Small size Low cost Fast switching Long lifetime Minimal bulb replacement Low heat generation Low energy consumption Robust	Low spectral resolution Sensitive to wide voltage fluctuations High junction temperature Low light intensity	[66]
Laser excitation	Emission of fluorescence and Raman Narrowband pulsed light	Composition detection at pixel level High intensity light Narrower bandwidth than LED Signals are not interfered by carbon or water absorption	Detection of weak Raman signals is challenging due to high-fluorescence background	[71]
Tuneable light source (Quartz–Tungsten Halogen lamp)	Near UV VIS NIR	Area scanning Weak illumination (using wavelength dispersion) reduces heat damage of samples	No point or line scanning	[72]

^1^ Low heat–load illumination is also available and provides an evenly distributed illumination line while emitting very low heat compared with the typical halogen lamp.

## 3. Image Acquisition Methods

Hyperspectral imaging collects information as images using well-defined spectral bands. The images are combined to form a three-dimensional (*x*, *y*, *λ*) hyperspectral data cube for processing and analysis, where *x* and *y* represent the two spatial dimensions of the scene and *λ* represents the spectral dimension (wavelengths) [64,73]. The resolution is characterized by the number of spectral bands of a range in the electromagnetic spectrum. Thus, multispectral images have low spectral resolution, given they have a maximum of 10 spectral bands, whereas HSI sensors have high resolution. A hyperspectral image comprises thousands or even millions of pixels. An ordinary image with 320 × 320 pixels contains 102,400 pixels. However, a hyperspectral image with 200 spectral bands has 20,480,000 pixels [74]. Spatial resolution, defined as the smallest distinguishable detail in an image [58], is also a factor; the size of the pixel or spatial point influences the signal-to-noise ratio [64,73].

Acquisition methods of a hyperspectral data cube include the following: (1) whisk-broom—one spectrum of a single point at a time; (2) push-broom—spectra of points of one spatial line at a time [75]; (3) tuneable filter—one waveband image at a time, much similar to a 2D photographic image (*x*, *y*); and (4) snapshot—full-waveband image at a time, much similar to capturing a photographic image with a third spectral dimension (*x*, *y*, *λ*). Push-broom is the most commonly used acquisition mode for applications in the food and agricultural industry. Push-broom imaging works either by the movement of the sample, for example, on a conveyer belt, or by directing the beam and detector to the region of interest [76].

Hyperspectral image acquisition is carried out in various sensing modes, including fluorescence [77], or one of four spectral modes typically utilized in the visible (400–700 nm) and NIR region—reflectance, absorbance, transmittance and interactance. These various imaging modes may be selected depending on the nature of the sample and the parameters being assessed. The light source for each optical mode is positioned differently to best capture the image based on the mode with minimal interference.

Hyperspectral Fluorescence Imaging (HSFI) detects chemical components that produce a fluorescence emission in the visible region (400–700 nm) when excited with short wavelengths (e.g., ultraviolet (UV) radiation or monochromatic laser light) [77]. These include components such as chlorophyll and some pigments in seed samples [78]. There are many examples of fluorescence imaging used in assessing physical and chemical quality parameters in food products [79], as well as faecal contamination in apples [80]. However, HSFI is unable to measure many of the food quality attributes that are detected by the optical modes (e.g., reflectance), such as soluble solid content, fruit pH and maturity discrimination [80].

Reflectance is the most common hyperspectral mode used in agriculture due to the ease in obtaining responses from higher light levels. Quality parameters such as size, shape, colour and surface defects are generally detected in reflectance mode [76,81].

Some applications show better calibration models and prediction outcomes in interactance and transmittance modes [82,83]. In transmittance mode, light is transmitted through the sample, with a detector opposite to the light source to capture the light that has passed through the sample; the strength is often weak and sample dependent but is considered to be a more valuable response in relation to internal components and defects [84]. In interactance mode, the light source and detector are located on the same side; however, the received light is sealed from the environment to prevent interference [85]. This particular mode is thought to be a combination of reflectance and transmittance as it can penetrate the sample, extracting more information than reflectance mode. The conformation of the light and detector is important to avoid refraction, specular reflectance and scattering in all three optical modes.

Another technique used in hyperspectral image acquisition is absorbance. Although there are limited reports of its use in agriculture, it is a preferred method for the quantitation of chemical constituents, for example, protein and oil contents of wheat grain [86].

## 4. Data Analysis Steps for Assessing Seed Quality

Given the enormous size of the image data acquired, effective pre- and post-processing methods are necessary to reduce data dimensionality by eliminating spurious signals and improving the classification models [74]. Spurious signals can include background and specular reflection regions, non-homogeneous illumination and abnormal pixels. Hyperspectral image processing typically involves three phases, (1) pre-processing; (2) analysis—calibration and prediction; (3) image processing.

There are many hyperspectral image processing tools that support the image processing of large data volumes (Table 2). The following describes the fundamental steps for processing any hyperspectral image:

(1) The raw spectral image acquired using hyperspectral imaging is initially corrected with a black and white reference image collected with the camera sensor. The image quality is subsequently processed to improve and enhance certain characteristics. These can include the more typical magnification, colouring, cropping and sharpening, as well as more complex noise reduction and image enhancements using the Fourier transform (FT) and wavelet transforms (WTs) under various methods [87]. The WT and FT can also be used as compression tools for the reduction in spatial and spectral information.
(2) Spectral pre-processing entails algorithms that correct noise and artifacts generated from light scattering, specular reflectance (mirror-like reflectance) and variation in surface morphology. This step is important in developing a robust model with reliable predictability. The most popular pre-processing algorithms include the following:
  - Smoothing can contribute to the removal of instrumental noise without reducing spectral resolution. The most common technique is the Savitzky–Golay approach; other methods include moving average, median filter, Gaussian filter, WT and principal component analysis (PCA) for outlier identification [69,87,88].
  - Light-scatter correction/minimisation can be achieved with multiplicative scatter correction (MSC), extended multiplicative scatter correction/signal correction (EMSC). Standard normal variate (SNV) is a row-oriented transformation which centres and scales individual spectra. These techniques are competent to reduce the spectral variability and baseline drifts across samples [89].
  - Derivatives (mainly first and second derivatives) are methods used to remove additive and/or multiplicative effects in spectral data [89]. The first derivative removes baseline drifts and the second derivative has the function of resolving linear trends and sharpening spectral features.
  - Orthogonal signal correction (OSC) achieves the removal of excessive background by filtering from the spectral matrix X, the component that is orthogonal to Y, i.e., it removes the uninformative component from the response variable Y [90]. This technique is used in conjunction with multivariate analyses such as constrained principal component analysis (CPCA) or partial least square regression (PLSR).
(3) Image segmentation is the ability to detect or discriminate objects or regions of interest (ROIs) from the image background. The most used and simplest segmentation algorithm is thresholding (e.g., PCA or wavelength channels) when a high contrast background material is used. This method works well when the background is uniform and contrasts the object or ROI. Images can be further processed using common morphological operations such as dilation and erosion. Dilation improves object visibility by adding pixels and erosion removes small pixels that are not part of the substantiative image. More advanced techniques such as deep-learning-based semantic segmentation methods (e.g., region-based segmentation) can be applied, providing pixel level recognition for the selection of ROIs [91].
(4) Subsequently, the huge magnitudes of pre-processed data are further analysed using multivariate analyses to identify the desired relationships of the acquired sample images based on the hyperspectral imaging data. Unsupervised methods: PCA, k-means clustering and hierarchical clustering. Supervised methods (predefined known classes): Typical supervised multivariate classification algorithms for the analysis of hyperspectral imaging data include linear discrimination analysis (LDA), partial least square–discriminant analysis (PLS-DA), support vector machines (SVM), k-nearest neighbour (kNN) and deep learning approaches based on artificial neural networks (ANNs) such as convolutional neural networks (CNNs) [92].
(5) Multivariate regression can be used to establish predictability by forming a relationship between the target features of the spectrum and their quantitative or qualitative response in the sample. Multivariate linear regression methods in quantitative analyses of spectral data mainly include multiple linear regression (MLR), principal component regression (PCR) and PLSR [93,94]. Non-linear regression techniques include ANNs [95] and support vector regression (SVR) [96,97]. ANNs simulate the behaviour of biological neural networks for learning and prediction purposes. LS-SVM, an optimized version of the standard SVM, is commonly used for spectral analyses.
(6) Effective wavelength (EW) selection: Hyperspectral imaging can also be classified as an exploratory analysis. Once the EWs are determined, data reduction and analysis speed can be achieved. For example, popular methods include partial least squares regression (PLSR) and stepwise regression (SWR). More sophisticated methods include the successive projections algorithm (SPA), genetic algorithm–partial least squares (GAPLS) [94] and interval partial least squares (iPLS) [98]. SPA and uninformative variable elimination (UVE) are two relatively sophisticated methods. UVE eliminates uninformative variables but its selected variables might have a problem of multicollinearity and SPA selects variables with minimal multicollinearity, but its selected variables might contain variables less related to the quality attribute. Thus, UVE-SPA was proposed by Ye, S. et al., 2008 [99], to complement the advantages of both methods and has been applied to the spectral analysis of food quality [100,101]. In addition, once the wavelengths of interest are known, multispectral imaging systems can be used to reduce costs, data storage and analysis requirements. Calibration models based on unique regions of the NIR spectrum that are informative are very important, because, as many other spectroscopic techniques, NIRS is also subject to interference signals from other components. To avoid significant loss of analytical precision and accuracy, effective wavelength selection methods coupled to full spectrum calibration techniques are paramount for the performance of calibration methods [102].
(7) Model evaluation outputs: Various cross validation techniques can be applied, including leave-one-out cross validation. Within the processes of calibration, validation and prediction, the performance of a calibration model is usually evaluated in the following terms: classification error cross validation (CV), root-mean-square error of calibration (RMSEC) and coefficients of determination (R^2^) of calibration (R^2^ Cal) in the calibration process; root-mean-square error of cross-validation (RMSECV) and coefficients of determination of cross validation (R^2^ CV) in the validation process; and classification error of prediction (CEP), root-mean-square error of prediction (RMSEP) and coefficients of determination of prediction (R^2^ Pred) in the prediction process. Generally, a good model should have higher values of R^2^ Cal, R^2^ CV and R^2^ Pred (>0.7), lower values of CV (<0.3), CEP (<0.3), RMSEC, RMSECV and RMSEP and a small difference between CV and CEP. The calibration models’ accuracy and reliability depend on the training data set. Increasing the replication or data in the training set increases the accuracy; however, reducing the variance and bias in the training set can improve overall predictability.
(8) Samples that are inherently non-homogenous, such as fruit and food products, can be accurately depicted using visualisation techniques of their hyperspectral images. The NIR-HSI not only provides morphological information such as other conventional cameras but also a high resolution spectral chemical fingerprint for each pixel in the image data acquired. Images of individual wavelengths can be displayed (e.g., videos can be generated in MATLAB’s image processing toolbox). This is usually beneficial when the intensities correlate to a wavelength that reflects an important property of the sample.

**Table 2:** Examples of image processing tools reported for NIR-HSI seed quality.

Image Processing Tools	Characteristics	Reference
MATLAB (The Math-Works Inc., Natick, MA, USA), e.g., image processing toolbox (IP); statistics and machine learning toolbox (STSMS); Pls_Toolbox/multivariate image analysis toolbox (MIA_Toolbox)	Development of algorithms and models Data analysis More flexible image visualisation and analysis than Environment for Visualising Images (ENVI) Built-in math functions Faster data analysis exploration time than traditional programming languages	[103,104]
Unscambler (CAMO, Norway)	Multivariate data analysis Data mining and calibration of spectral data	[105]
ENVI software (Research Systems Inc., Boulder, CO, USA),	Image processing, analysis and display using tailored algorithms Wizard-based approaches and automated workflows for user-friendly image processing	[61]

To achieve high accuracy for the classification of samples in NIRS, the sample size is higher than that needed for other analytical techniques. This is primarily because absorption in the NIR region is relatively weak and is typically used in reflectance mode. The higher the sample size, the greater the performance of the calibration and prediction models. Sample sizes for seed applications can be well over 1500 samples.

## 5. NIRS-Based Imaging Applications for the Detection of Chemical Components in Seeds

NIR-HSI has gained a lot of momentum in the last decade and there is an emphasis on developing deep statistical models for various applications. However, molecular interpretation is challenging and this is mostly due to the broad and overlapping signals represented in NIR spectra compared to its MIR spectroscopic counterpart. Mid-infrared (MIR) spectroscopy reveals characteristic frequencies that enable ease of band assignment and interpretation. There are also many databases for the interpretation of these bands, making MIR spectroscopy very useful for the qualitative analysis and interpretation of chemical components. NIR spectra are weak and dominated by overtone and combination bands of C-H, O-H and N-H functionalities. Intense absorptions of C=O and C-O are normally found in the MIR region and are rarely represented in NIR [106]. However, NIRS is unique as it relies on the chemical fingerprints of matrices as opposed to the structural compound interpretations used in MIR spectroscopy. The fingerprint regions of the NIR make it useful for classification using powerful computerized data processing techniques for the quantitative interpretation of the complicated NIR spectra. The technique is also known as chemical imaging, as it provides information at the molecular level. Absorbance at the NIR frequency bandwidth reflects overtones of C-H, C-O, O-H and N-H stretching vibrations [106,107,108,109,110,111,112,113,114,115,116,117].

NIRS chemical band interpretation in NIR-HSI applications for the quantitative analysis of moisture, protein, starch, lipid and fibre content in grain and other seeds is routinely performed to support wavelength selection and calibration models (Table 3). In machine learning models, interpretation can often be difficult, leading to uninterpretable “black box” calibration algorithms. There are numerous NIR-HSI calibration models that do not corroborate the model with interpretation data [104,118,119], which could lead to real-time application errors. In recent years, heatmap-based visualization methods that unravel the internal mechanisms of deep learning models have been developed. Although there are a number of proposed methodologies, the class activation mapping (CAM) methods (e.g., GRAD-CAM) are becoming increasingly popular [120].

There are subtle differences in the overtone and combination bands associated with the same chemical components in different sample types for, e.g., the C-H and O-H stretches in oils [112] plant material [121] and in seeds [122] (Table 3) are intrinsically different. Chemical bands, particularly for oil and fatty acids, also vary within seed types, as NIR-HSI instruments are sensitive to analyte morphology and composition. The variation is attributed to the vibrations within inherent molecules rather than changes in the C-H vibrations, such as the C-H stretches in carbohydrates differ from those in fatty acids (Table 3). Overall, characteristic wavelengths indicate that the fundamental shifts are consistent in both oil [112] and plant material [121], despite their overall chemical composition, although discrepancies may occur due to overlapping signals from other chemical components.

The major components responsible for the absorbance bands in the NIR region are primarily due to C-H bonds, mainly from fats and oils; O-H bonds, mostly associated with water; and N-H bonds, primarily found in protein. NIRS information of fatty acids is distributed in several regions along the NIRS spectrum. The 1710 nm band has been assigned as the first overtone of the asymmetric C-H stretch in methyl or methylene groups and associated with fatty acids [81,107,115,123,124]. The 1985 nm band is most likely known for protein absorption at 1980 nm related to the asymmetric combination of N-H [125,126]. Interferences from water absorptions could cause variations mostly due to shifts associated with hydrogen bonding [81].

These unique spectral bands have been utilized in NIRS and proven to be effective as a quantitative technique, as demonstrated by the numerous reports that indicate successful calibration methods for the quantitative analysis of fatty acids, oils, carbohydrates, proteins and many chemical components (Table 1) [106,107,108,109,110,111,112,113,114,115,116,117,122,127,128,129,130]. There are also measurements of chlorophyll, erucic acid (carcinogen) and glucosinolate (toxin) in canola [109]. However, NIRS is reliable in estimating oil and fatty acid content in processed seed such as oil and powder [116,128]. Intact seeds are generally found to be less accurate than processed seeds; however, it may still be used [116,128]. Other examples in NIR technology have been used to detect plant leaf water stress in the region 950–970 nm [131,132]. In addition to the wavelength 970 nm for leaf water stress, wavelengths of 870 nm, 910 nm, 936 nm and 950 nm exhibited different water absorbance patterns for virus induced hypersensitivity water stress [131,132], which was used to confirm soybean mosaic infection. The response at wavelength 936 nm influences NIR spectra though the hydrogen-bonded O-H stretch that occurs in protein–starch complexes [131,133].

These fundamental quantitative applications in NIRS have also been effective in NIR-HSI techniques. For example, calibration models were established to quantitate moisture [134], oil [135], anthocyanins [136] and fungal contamination in fruit using quantitative visualization methods [137].

**Table 3:** Visible and NIR band assignments associated with seed composition and viability, as well as bacterial and insect infestation in selected examples.

Seed Sample	Wavelengths (nm)	Vibration	Chemical Component	Characterisation	Reference
Corn	1210 and 1460; 1724 and 1760; 2058	C-H second overtone; first overtone vibration -CH_2_ and -CH; N-H combination band	Carbohydrate Carbohydrate protein	Viability	[138]
Watermelon seed	479 (blue), 517 and 565 (green), 717 (red); 832; 913 and 985 nm	Blue, green and red bands; C-H combination band; -OH	Visible/colour differences; fat; bacterial effect on composition associated with water stress	Bacterial infestation	[103]
Norway spruce (*Picea abies*)	1710; 1985; 1450 and 1940; 2090;	First overtone of asymmetric C-H stretch; asymmetric combination of N-H broad first overtone; first overtone and combination bands of -OH; C-H stretch	Fatty acid; protein; water/starch/cellulose; carbohydrate from starch and cellulose	Viability Bacterial infestation Empty seeds	[139]
Basil seed (*Ocimum basilicum* L.) origin	1449–1457; 1242–1254; 1380 and 1696	First overtone of -OH; C-H second overtone; -OH stretch; first overtone of asymmetric C-H stretch	Water; crude lipid; total phenolics; fatty acids	Seed origin	[118]

## 6. Hyperspectral Imaging in Agriculturally Important Seeds

The discrimination among seed varieties and checking seed purity play a key role in plant breeding programs, seed production, gene-bank management and in the general trading of seeds. Testing seed purity involves checking for the presence of plant debris, foreign materials, weed seeds, contaminating species and broken and damaged seeds. Separating varieties and determining their critical properties in terms of DUS (distinctness, uniformity and stability) are significant for variety registration and intellectual property rights of plant breeders, as well as for developing new varieties in the market place [140].

Seed quality is traditionally defined by seed health, as well as varietal purity and physiological or nutritional characteristics. Pathogen-infected seeds can be evaluated in numerous ways, depending on the organism being tested, but, generally, all methods are time consuming or expensive, such as visual examination of seeds, polymerase chain reaction (PCR) tests and immunoassays [141,142]. The most sensitive tests, such as PCR, are not only complicated and expensive but destructive; therefore, an evaluation of the batch can only be performed in a random subset of seeds from each batch.

Seed physiology is evaluated by germination tests alongside seed vigour evaluation [143], as germination tests may not provide a true picture of seed potential [140]; seed vigour is typically an indicator of the performance of the seed outside the optimal conditions. There are automated systems available to make measurements of the growth of the seedlings to determine the seed potential. However, assessing seed quality using destructive sampling is not ideal, particularly when seeds are limited or valuable. Seed quality assessments are also labour intensive and time consuming. The improvement of seed quality analyses has been recognized by organisations that develop standardised methods for seed quality analysis, such as International Seed Testing Association (ISTA) and Association of Official Seed Analysts (AOSA) [144].

The use of sophisticated imaging technologies in agriculture has transformed the quality control process with minimal human intervention. Initial applications of image processing to the food and agricultural products were the use of RGB (Red–Green–Blue) colour vision systems for grading and identifying defects [145]. Although useful for surface defects, RGB is incapable of detecting contaminants and trace constituents. The use of hyperspectral cameras is increasing in phenotyping applications, as they allow physiological responses, pathologies, or pests to be identified in a non-invasive way. Although there is an increase in phenomic setups using multispectral and hyperspectral cameras, significant investments would be required to develop and deploy systems with the necessary computing infrastructure, power and storage.

The NIR-HSI is an opportune technological advancement, with demonstrated success in seed quality assessment, now viewed as a necessary alternative to measuring individual seed quality, cultivar purity and viability in a non-destructive, high-throughput manner.

## 7. Opportunities for Lolium Species—Establishing a Pipeline for Seed Phenomics

Given the various agricultural applications, we investigated the potential of using this technology for *Lolium* spp. Here, we describe a generic imaging pipeline for assessing seed quality characteristics in perennial ryegrass (*Lolium perenne*) that could also be applied to other *Lolium* species, such as tall fescue (*Lolium arundinaceum*). The established pipeline also provides a basis for exploring more exhaustive chemometric and acquisition optimization parameters.

### 7.1. Perennial Ryegrass Seeds

Perennial ryegrass is a commonly sown temperate grass used for pasture and turf throughout the world, with high economic importance as a forage in Australia and New Zealand [146]. Asexual endophytic *Epichloë* spp. (henceforth referred to as endophytes) establish a symbiotic relationship with perennial ryegrass [146]. These interactions are beneficial to the pastoral agriculture industry, as the endophyte confers resistance to biotic and abiotic stresses [146]. The beneficial endophyte, therefore, plays a major role in the marketing of perennial ryegrass seeds [146]. Asexual endophytes are characterised by vertical transmission, inherited via the seed, of the fungal hyphae. However, the vertical transmission of endophytes to seed is not a perfect process, in that the endophyte is not necessarily transmitted to all seed and sometimes with poor viability. There are several seed quality attributes that are typically tested in perennial ryegrass seeds. These include seed viability (germination), endophyte presence and strain identity, as well as endophyte viability. The current techniques of germination and PCR-based assays for viability or endophyte detection are expensive and destructive, as large numbers of seeds are tested for better representation of seed quality of a batch.

### 7.2. Spectral Acquisition Parameters

**Figure 1:**
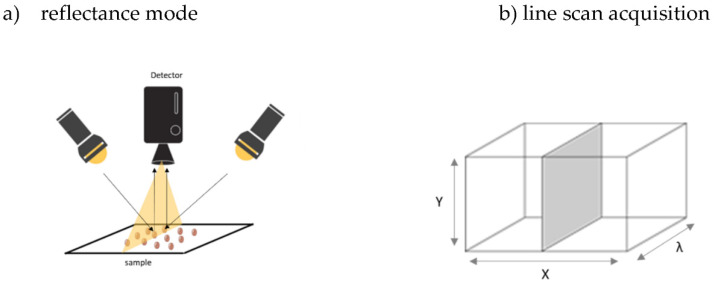
A representation of seeds laid on a platform and acquired in (**a**) reflectance mode with (**b**) line scan (push-broom) scanning (X, Y spatial and λ spectral dimensions); the filled grey box indicates the image acquired at each time.

In reflectance mode, a line scanning (push-broom method) is selected to acquire HSI images (Figure 1). An NIR spectrum is averaged for each pixel and captured across each spatial line. A complete spectral image is obtained by movement of the sensor across the sample. The spatial resolution can be adjusted by parameters such as frame rate and camera height (field of view). Obtaining high spatial resolution data can lead to difficulties in data processing. Thus, data acquisition parameters need to complement data processing capabilities.

**Figure 2:**
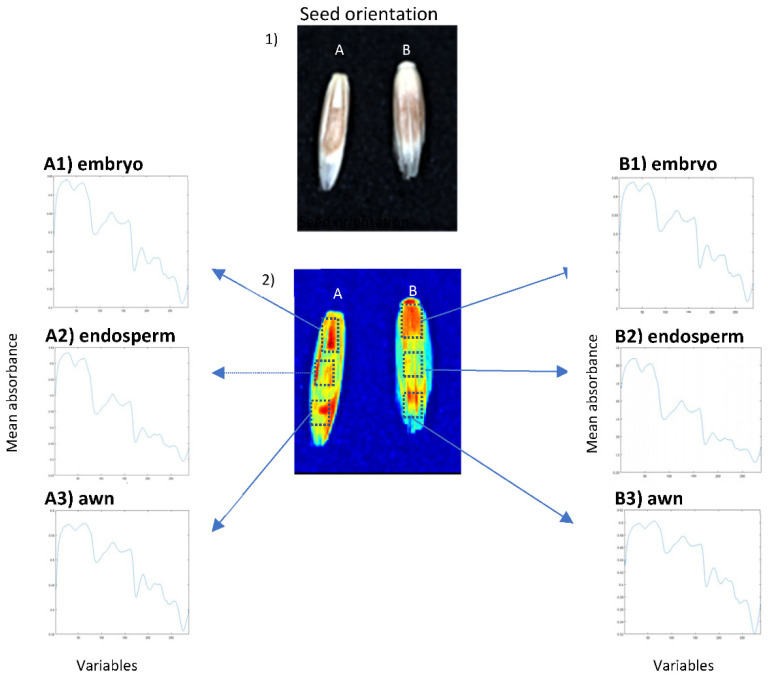
Image of perennial ryegrass seed (1) and NIR-HSI image (2) showing seed orientations: embryo facing up (**A**) and embryo facing down (**B**). The two orientations showing mean absorbance of raw normalised spectra of the embryo (**A1**), endosperm (**A2**) and awn (**A3**) regions of embryo facing up and embryo (**B1**), endosperm (**B2**) and awn (**B3**) regions of embryo facing down. The variables represent 288 wavelengths acquired in the SW-NIR reflectance (1000–2500 nm) region.

The morphology of perennial ryegrass seeds varies on both sides, making it possible that one orientation may be favoured over the other in a calibration model. However, preliminary investigations showed that both orientations had only subtle differences in profile across the key regions of the seed, including the embryo, endosperm and awn (Figure 2). The ideal surface for hyperspectral imaging is one with a flat surface, as asymmetric samples can cause variations in the spectra obtained due to scattering of incident light. This is important to consider and may be overcome with large numbers of sample replicates [75]. When reflectance mode is applied, there is very little penetration of light. The penetration depth is often negligible in samples with high opacity and complexity; thus, by no means, should a hyperspectral image be considered representative of the entire composition of a sample [88].

### 7.3. Sample Preparation for Seed Classification Methods

**Figure 3:**
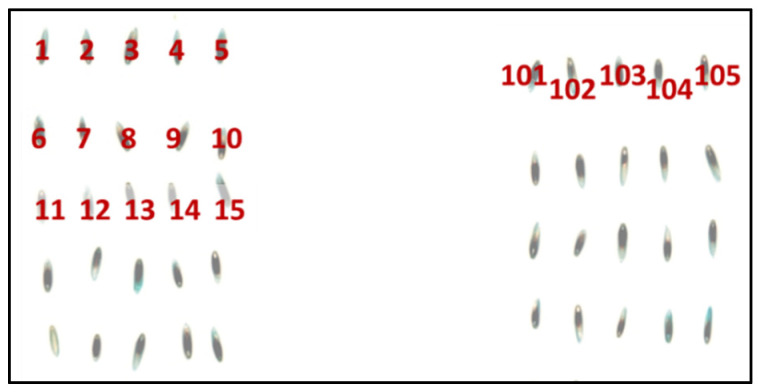
Seeds laid in a grid format for NIR-HSI acquisition to allow individual seed tracking to be conducted.

Typically, with hyperspectral imaging techniques, many replicates are required to build a classification model and this could range from 300 to 13,000 individual seeds (Table 4). Seeds are generally placed on a black or dark substrate in a grid format, so individual seeds can be tracked and assessed for properties such as viability or endophyte infection (Figure 3). A dark substrate is chosen for zero reflectance in the NIR and SW-NIR regions of the spectrum and it allows the background to be easily distinguished from the seeds using class discrimination models in any imaging software (Table 2). Light or white substrates saturate the sensor in the NIR region, making it difficult to discriminate using class discrimination models. Once the data are acquired, seeds can be accurately tracked throughout seed quality testing (e.g., seed and endophyte viability) using current industry standards.

### 7.4. Data Analysis Pipeline Using the MIA_Toolbox Add-On for PLS_Toolbox

**Figure 4:**
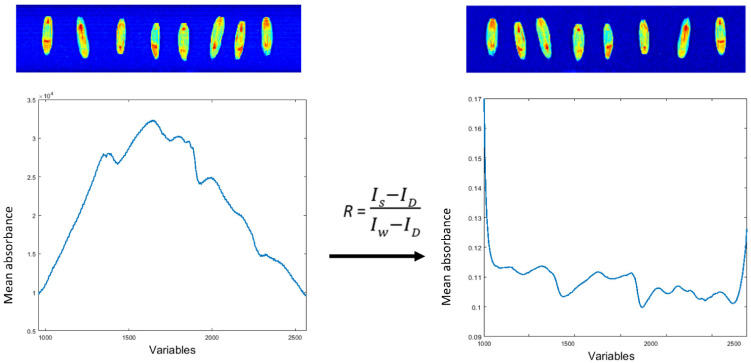
An example of an average spectrum of perennial ryegrass seeds pre- and post-white-and-dark calibration. The variables represent 288 wavelengths acquired in the SW-NIR reflectance (1000–2500 nm) region.

**Figure 5:**
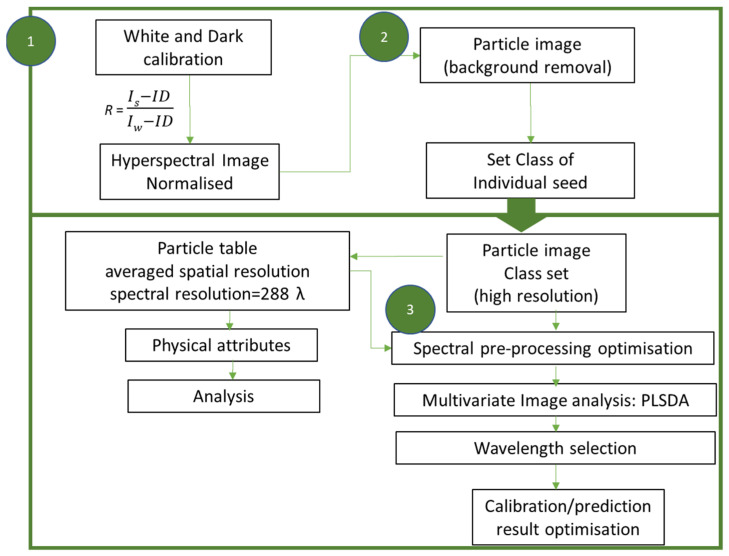
Flow chart of a series of steps for analysing hyperspectral image data of seeds in MIA_Toolbox. (1) Hyperspectral image normalisation. (2) Background removal and class selection of individual seeds. (3) Particle image/particle table spectral pre-processing optimisation and calibration and prediction.

The raw hyperspectral images acquired by either a visible or near-infrared imaging camera are corrected by the white and dark reference images to obtain spectral reflectance values [74] (Figure 4). This may be performed via image processing tools or the MATLAB script editor. The normalised image can be processed in MIA_Toolbox as a two-dimensional data set (Figure 5). The image manager tool is used to crop images to generate a binary image to compute the class separation of seeds and background in the particle analysis tool using a reverse-mask option, contrasting seeds from the background. A particle image and particle table can be generated here and used to perform multivariate analyses. The particle table is an average NIR reflectance spectrum from the whole sample surface. The particle image provides reflectance spectra at each pixel (Figure 4), utilising the full spatial distribution of molecular vibration information for each seed that increases the depth of the data and can assist in selecting regions with greater predictability [139]. The high spatial resolution of individual seeds may pose challenges for high-throughput screening, as well as data processing. Here, the individual seeds can be selected and classified into respective groups based on the classification model. The particle tables are smaller in file size and include information on the physical attributes of the object, such as roundness and size.

The pre-processing methods for the removal of undesired effects that correct for noise, light scattering and specular reflectance from variation in surface morphology are used to improve the calibration model performance. The recommended pre-processing method for NIR-HSI perennial ryegrass average absorbance data (particle table) includes detrending to remove the mean offset from each sample (row), extended multiplicative scatter correction/signal correction (EMSC), OSC filter, Savitzky–Golay smoothing and derivatives and mean centre to remove mean offset from each variable. The commonly utilised pre-processing method for high spatial resolution data (particle image) is 2nd derivative, standard normal variate (SNV) and a mean centre to remove the mean offset from each variable. The particle image data contain enormous numbers of data and complex pre-processing is not recommended.

High-dimensional imaging data can be subjected to two approaches of multivariate analysis, including unsupervised and supervised classification. Unsupervised techniques that require no class information of the data include PCA. PCA is also an effective method to detect and remove outliers. The supervised classification methods include linear methods such as PLSDA or nonlinear approaches such as the support vector machine (SVM), common techniques for analysing full-wavelength data NIR-HSI data (Table 2). The next step is to select candidate wavelengths with variable selection tools based on variable importance projection (VIP) and selectivity ratio (sRatio). If the model separates in a PCA score plot, a loading plot can be generated to evaluate the wavelengths that give a significant contribution to the classification model. Those that possess a high loading value could be selected and evaluated. The full-wavelength and EW selection can be evaluated for calibration, validation and prediction performance using the R^2^ Cal, R^2^ CV, R^2^ Pred, CV, CEP, RMSEC, RMSECV and RMSEP numerical parameters. A wavelength selection technique is highly recommended to develop a parsimonious model that can easily be transferable to low-cost online multispectral systems. The selection of a particular series of methods also depends on the classification challenge, the size of the data set, the implementation and economic practicality.

There are limited reports on the online application of NIR-HSI for seed quality attributes. Some examples include determining the age of wheat seeds in a real-time application using the GaiaSorter Hyperspectral Sorting System with calibration and test data acquired directly from the system [61]. The transference of calibration models developed on NIR-HSI small-scale lab scanner systems to industrial applications can lead to losses in accuracy related to the random distribution of the samples on a conveyor belt system, increased speeds and lighting variations in the visible range of the electromagnetic spectrum, for example, in sorting adulterated almonds [60]. Therefore, the hyperspectral imaging development for applications that would eventually be transferred to a seed sorting device requires a parsimonious calibration model to reduce the high-dimensional spectral and spatial computational burden using the afore-mentioned EW selection algorithms. The use of EW selection allows low-cost multi-spectral sensors to be used in place of hyperspectral cameras, further reducing deployment costs.

**Table 4:** Selected applications of NIR-hyperspectral imaging in agriculturally important seeds (reflectance mode).

Application	Classification Methods	Instrument Spectral Range and Wavelength Selection	Wavelength Selection/Full-Wavelength Range	HSI System Software	Data Processing Software	Calibration/Training and Prediction/Test Set Accuracies	Reference
Detection of bacteria-infected watermelon seeds (n = 336)	PLS-DA and least-squares support vector machine (LS-SVM)	400–1000 nm; visible and near infrared hyperspectral imaging (VNIR HSI); spectral resolution, 46 nm; spatial resolution, 0.22 mm	Wavelength selection based on RMSEV values (493–584 nm and 684–1004 nm) and full wavelength using PLS-DA classification were comparable	Visual Basic 6.0	MATLAB	FW: PLSDA calibration and prediction accuracy of 91.7% WS: PLSDA calibration of 91.3% and prediction of 90.5% accuracy	[103]
Classification of glycyrrhiza seeds (planting pattern, species and origin) (n = 475)	PLS-DA; SVM	948–2512 nm; spectral resolution, 5.45 nm	Wavelength selection based on PCA using SVM classification was superior compared to full wavelength using PLS-DA	ENVI5.3	MATLAB R2017b	*Planting pattern*: PLS-DA calibration = 100% and prediction = 92.83%; SVM calibration = 98.22; test = 96.97% *Species*: PLS-DA calibration = 95.56% and prediction = 100%; SVM calibration = 99.56%; prediction = 97.75% *Seed origin*: PLS-DA calibration = 100% and prediction = 86.11%; SVM calibration = 97.75% and prediction = 93.67%	[104]
Classification of Norway spruce (*Picea abies*) viable seeds, empty seeds and seeds infested by *Megastigmus* sp. larvae. (n = 1606)	Support vector machine (nu-SVM) and sparse logistic regression-based feature selection	Short-wave infrared (SWIR; 1000–2500 nm range)	Wavelength selection using logistic regression using nu-SVM classification model	-	MATLAB 7.9 and LIBSVM (“nu-SVM” classifier)	Leave-one-out classification accuracy: for WS, 93.8% (3 wavelengths) and 99% (21 wavelengths); for FW, 99.2% accuracy	[139]
Discrimination of basil seed (*Ocimum basilicum* L.) origin (Singapore, India, Pakistan or Vietnam) (n = 480)	PLS-DA (calibration)	900–1700 nm; spectral resolution = 5 nm	-	Microsoft Windows Operating System	Unscrambler (v10.5)	Full wavelength: calibration = 90.12% prediction = 88.19%	[147]
Cotton seed varieties (n = 13,160)	PLS-DA; LR; SVM	942–1646 nm	Effective wavelength selection: PCA	-	Deep learning (CNN, ResNet); PLS-DA; SVM; LR	Full wavelength: CNN and ResNet with (LR/Softmax/PLS-DA/SVM) calibration of 91–99%, validation of 84–89% and prediction of 82–88%. Selected wavelength: CNN and ResNet with (LR/Softmax/PLS-DA/SVM) calibration of 87–98%, validation of 76–84% and prediction of 75–84%	[118]
Maize seed varietal classification (n = 1632)	PCA; LS-SVM	400–1000 nm	Wavelength selections: multi-linear discriminant analysis (MLDA) vs. UVE and SPA	ENVI 4.3	MATLAB 2009b (LS-SVM toolbox)	Full wavelength: calibration of 100% and prediction of 93.26%. Wavelengths, 5–15 selected: MLDA calibration of 99.39–99.88% and prediction of 90.40–93.81%; UVE calibration of 99.46–99.85% and prediction of 88.20–91.94%; SPA: calibration of 98.66–99.40% and prediction of 80.8–87.40%	[119]

## 8. Conclusions

The burgeoning field of hyperspectral imaging for seed phenomics is fast becoming a promising cutting-edge technological advancement for the assessment of seed adulterations, microbial infections, morphology, varietal purity, age and quantitative nutritional properties. Although the capability of the chemical imaging technique is not without its challenges, it is a much-needed necessity, as current quality control measures for the inspection of seeds are expensive and labour intensive. There is an extraordinary number of reports on the capabilities of NIR-HSI technology and its real-time applications, particularly for the assessment of food quality. However, seed phenotyping can be more challenging, as physiological properties may not be as obvious when using imaging techniques due to constraints in size and the quantities of individual seeds required to be evaluated in a high-throughput manner.

In this review, we provide an NIR-HSI workflow and data-analysis pipeline for *Lolium* spp., using perennial ryegrass seeds, which reveals both challenges and recommendations in developing classification models. The review also explores and reports the wide scope of techniques utilized in NIR-HSI imaging for seed phenomics. Subsequently, providing alternative measures to further develop NIR-HSI pipelines to explore more complex physiological traits in seed that should be explored in future research efforts.

There are very few reports of real-world applications of NIR-HSI seed quality assessments; thus, it is anticipated that on-going improvements in chemometrics, imaging toolboxes and technological advancements will drive the deployment of on-line automated systems for seed sorting in the future.

## Data Availability

Data presented in this review is contained within the article.
